# Novel green tissue-specific synthetic promoters and *cis*-regulatory elements in rice

**DOI:** 10.1038/srep18256

**Published:** 2015-12-11

**Authors:** Rui Wang, Menglin Zhu, Rongjian Ye, Zuoxiong Liu, Fei Zhou, Hao Chen, Yongjun Lin

**Affiliations:** 1 National Key Laboratory of Crop Genetic Improvement and National Centre of Plant Gene Research, Huazhong Agricultural University, Wuhan, China; 2 College of Foreign Language, Huazhong Agricultural University, Wuhan, China

## Abstract

As an important part of synthetic biology, synthetic promoter has gradually become a hotspot in current biology. The purposes of the present study were to synthesize green tissue-specific promoters and to discover green tissue-specific *cis*-elements. We first assembled several regulatory sequences related to tissue-specific expression in different combinations, aiming to obtain novel green tissue-specific synthetic promoters. GUS assays of the transgenic plants indicated 5 synthetic promoters showed green tissue-specific expression patterns and different expression efficiencies in various tissues. Subsequently, we scanned and counted the *cis*-elements in different tissue-specific promoters based on the plant *cis*-elements database PLACE and the rice cDNA microarray database CREP for green tissue-specific *cis*-element discovery, resulting in 10 potential *cis*-elements. The flanking sequence of one potential core element (GEAT) was predicted by bioinformatics. Then, the combination of GEAT and its flanking sequence was functionally identified with synthetic promoter. GUS assays of the transgenic plants proved its green tissue-specificity. Furthermore, the function of GEAT flanking sequence was analyzed in detail with site-directed mutagenesis. Our study provides an example for the synthesis of rice tissue-specific promoters and develops a feasible method for screening and functional identification of tissue-specific *cis*-elements with their flanking sequences at the genome-wide level in rice.

Synthetic biology, which combines biology and engineering to design and construct new biological parts, devices and systems, has shown great application potentials in many fields^1^, such as the creation of cells controlled by synthetic genomes^2^, acquisition of biofuels and pharmaceuticals produced by synthetic gene circuits^3^, and induction of the precise expression of target genes driven by synthetic promoters^4^,^5^. As an important part of synthetic biology, synthetic promoter has gradually become a hotspot in current biology^6^.

As the most important tool of gene expression regulation in genetic engineering and synthetic biology, promoters can precisely regulate the expression of target genes to expected patterns and levels^7^,^8^ and are also crucial for exploring the mechanism of transcriptional regulation^7^,^9^,^10^,^11^. More and more studies of synthetic promoters have been reported with the development of synthetic biology. Great efforts have been made to construct synthetic promoters in microorganisms. The major strategy is to fuse numerous *cis*-elements or random sequences with the core promoters and subsequently screen the synthetic promoters whose expression efficiencies are suitable for experimental purposes^12^,^13^,^14^. However, this approach is both labor- and time-consuming for the study of synthetic promoters in plants, especially in crops with long growth periods, such as rice. In animals, recent studies have been focused on combining different promoters to construct synthetic promoter with higher expression efficiency and specificity^15^,^16^.

Studies of synthetic promoters in plants are fewer and are mainly based on fusing *cis*-elements with core promoters. In 2012, Koschmann *et al.* applied BEST to discover conserved sequence motifs in the promoters of Arabidopsis genes up-regulated by multiple pathogenic stimuli. Then, they compared these motifs with the known *cis*-regulatory sequences in AthaMap, PLACE and AGRIS databases. The sequences showing no or only low similarity to the already-described regulatory sequences were analyzed with synthetic promoters^17^. This study provides an enlightening approach for seeking and identifying inducible *cis*-elements in other species. Another important work was conducted by Liu *et al.* in 2013, in which pathogen/defense signaling inducible *cis*-elements were fused with cauliflower mosaic virus (CaMV) *35S*/Minimal CaMV *35S* to drive a fluorescent protein reporter gene in stable transgenic tobacco and Arabidopsis. The expression of the reporter gene in transgenic plants with bacterial pathogens or phytohormone treatments demonstrated that inducible synthetic promoters can function in transgenic tobacco and Arabidopsis^5^. A recent progress of synthetic promoters achieved by this team is that they conducted a comprehensive bioinformatic analysis for *de novo* soybean cyst nematode-inducible motif discovery in the soybean genome and then applied synthetic promoters to identify the candidate motifs in transgenic soybean hairy roots^18^. This study proves that the new high-throughput screening method has a high application potential in the discovery of inducible *cis*-elements. Much work in plants has been focused on inducible synthetic promoters, while studies of tissue-specific synthetic promoters have been rarely reported, and there is still no report of any tissue-specific synthetic promoter in rice. The main reason is that few *cis*-elements with clear functions which are involved in tissue-specific expression have been reported and no effective method for screening these *cis*-elements has been developed. Therefore, a high-throughput method for screening and identifying *cis*-elements related to tissue-specific expression is critical to the development of tissue-specific synthetic promoters.

Rice is one of the most important food crops in the world and a model plant for functional genomic researches in cereals^19^. More complete genomic information^20^,^21^,^22^ and more explicit gene expression information^23^ greatly facilitate the studies of tissue-specific promoters. Much work has been done to clone tissue-specific promoters and to use them in genetic improvement of rice and gene functional analysis^24^,^25^,^26^,^27^,^28^. The above studies lay a solid foundation for seeking tissue-specific *cis*-elements and constructing tissue-specific synthetic promoters in rice.

In this study, we assembled several short promoters and *cis*-elements related to tissue-specific expression (*P*_*D540-544*_, *P*_*Osrbcs-550*_, *P*_*Osrbcs-62*_, *EnP3-110*, G box and GT1) as well as the first intron of rice *Act1* in different combinations, resulting in 5 novel green tissue-specific synthetic promoters which showed different expression efficiencies in various green tissues. Meanwhile, the functions of these expression regulatory sequences in synthetic promoters were also analyzed. As the feasibility of synthesizing tissue-specific promoters in rice was proved, an available method for the discovery of tissue-specific *cis*-elements is important to the development of tissue-specific synthetic promoters. Subsequently, we scanned and counted the *cis*-elements in different tissue-specific promoters based on the plant *cis*-elements database PLACE and the rice cDNA microarray database CREP, resulting in 10 *cis*-elements whose frequencies in green tissue-specific promoters were relatively higher. Finally, we identified a general regulatory sequence 5′-AAAATATTTAT-3′ (the underlined sequence indicates the core element), which can be applied in the synthesis of green tissue-specific promoters. As flanking sequence may influence the activity of the core element^17^, we applied site-directed mutagenesis to further analyze the function of the flanking sequence in detail. Our study provides an example for developing tissue-specific synthetic promoters in rice, and proposes a feasible method for screening and functional identification of tissue-specific *cis*-elements with their flanking sequences at the genome-wide level in rice.

## Results

### Creation of 5 novel green tissue-specific synthetic promoters (*GSSPs*)

**Figure 1:**
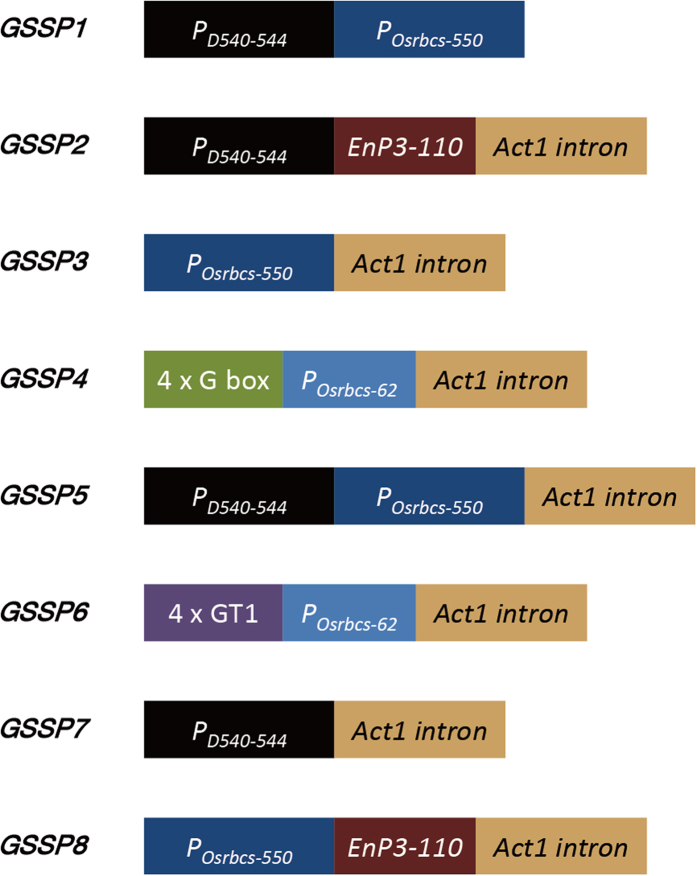
Schemes of 8 synthetic promoters assembled with different expression regulatory sequences.

Several regulatory sequences (*P*_*D540-544*_, *P*_*Osrbcs-550*_, *P*_*Osrbcs-62*_, *EnP3-110*, the first intron of rice *Act1*, G box and GT1) (Supplementary Fig. S1) related to tissue-specific expression according to previous reports^7^,^29^,^30^,^31^,^32^,^33^ were used for designing synthetic promoters. As the first intron of rice *Act1* is not a promoter but can increase the activity of the promoters adjacent to its upstream^29^, it was placed in the 3′ region of the synthetic promoters. Considering that the promoter module in the 3′ region of the combinations might be more important to its expression specificity and *P*_*D540-544*_ has activity not only in green tissues but also in root^7^, *P*_*D540-544*_ was placed away from the 3′ region of the synthetic promoters. If a single *cis*-element is added in the combinations, it should be quadrupled to increase its effect and placed upstream of a promoter, which is better to be a minimal promoter to avoid the interference from numerous *cis*-elements^5^,^17^. Therefore, G Box or GT1 was quadrupled and placed upstream of *P*_*Osrbcs-62*_ minimal promoter as the schemes of *GSSP4* or *GSSP6* showed (Fig. 1). Meanwhile, the specific combinations were designed for creating new synthetic promoters with desirable expression efficiencies and analyzing the functions of the regulatory sequences in synthetic promoters as well. *GSSP1, GSSP3, GSSP5* and *GSSP7* were designed to analyze the functions of *P*_*D540-544*_, *P*_*Osrbcs-550*_ and the first intron of rice *Act1* by comparing the activities of these synthetic promoters simultaneously. Likewise, *GSSP2* and *GSSP8* were selected to discover the function of *EnP3-110* by comparing the activities of *GSSP2* and *GSSP7* as well as *GSSP8* and *GSSP3*. The schemes of all the synthetic promoters are shown in Fig. 1.

**Figure 2:**
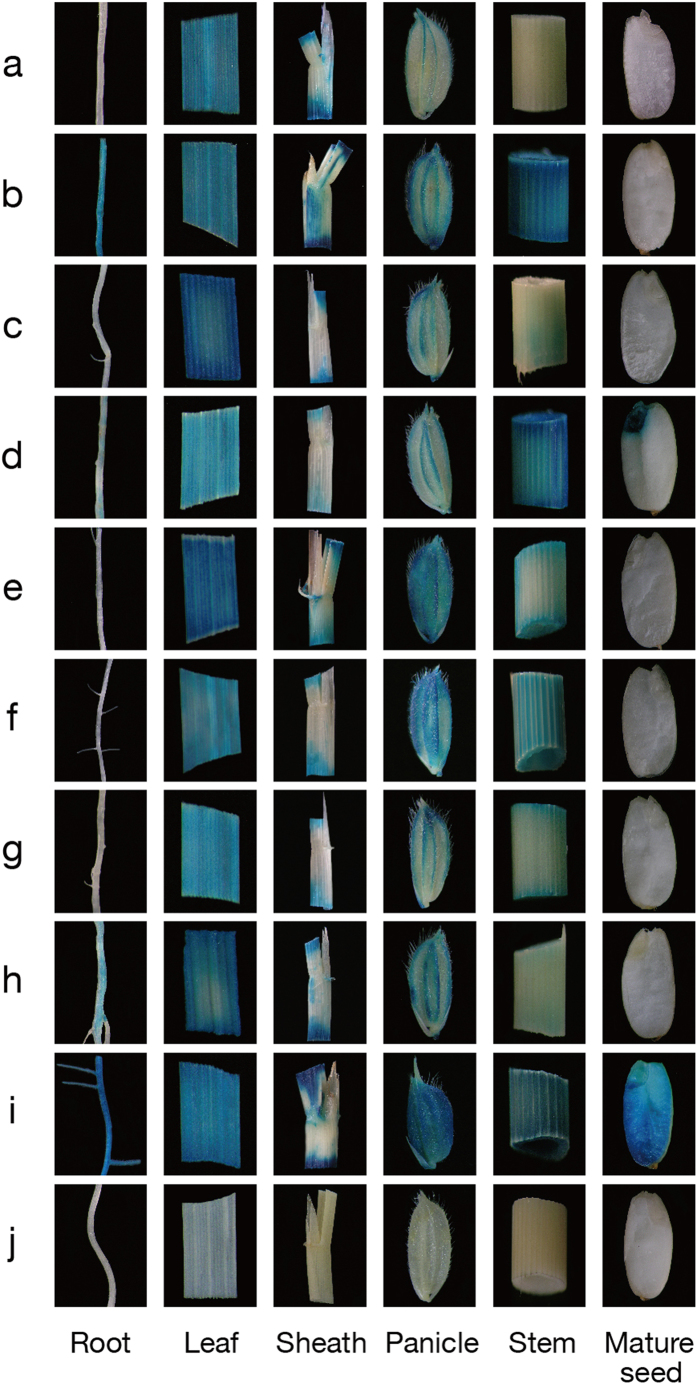
Histochemical analysis of *GUS* expression in various tissues of the transgenic plants containing different synthetic promoters/*GUS* fusions. (a–h) plants containing GSSP1-*GSSP8::GUS*; (i) positive control, plants containing CaMV *35S::GUS*; (j) negative control, plants containing pDX2181 empty vector.

**Figure 3:**
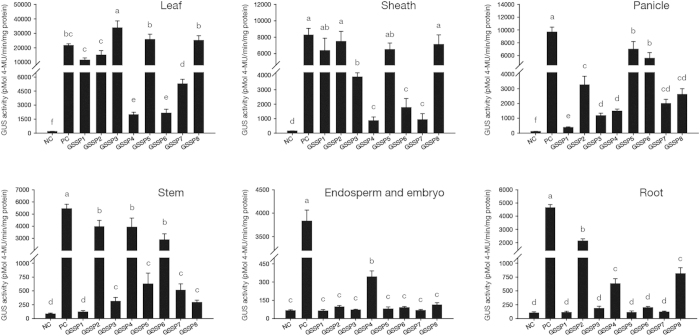
Quantitative analysis of GUS activity in various tissues of the transgenic plants containing different synthetic promoters/*GUS* fusions. *NC*, negative control, plants containing pDX2181 empty vector; *PC*, positive control, plants containing CaMV *35S::GUS*; (a–f ): significant difference (P < 0.05). Error bars indicate _SE_ based on five independent biological replicates.

After GUS assays in transgenic plants, the results of histochemical staining in different tissues showed that 5 novel green tissue-specific synthetic promoters (*GSSP1, GSSP3, GSSP5, GSSP6, GSSP7*) were obtained, which only showed expression efficiencies in all or some of the 4 green tissues: leaf, sheath, panicle and stem (Fig. 2). The GUS fluorometric activities of various tissues in *GSSPs* transgenic plants (Fig. 3) were in accordance with the histochemical staining results. The expression efficiencies of the 5 synthetic promoters in leaf were ranked as *GSSP3* > *GSSP5* > *GSSP1* > *GSSP7* > *GSSP6*; while in sheath, they were ranked as *GSSP1* > *GSSP5* > *GSSP3* > *GSSP6* > *GSSP7*; in panicle, they were ranked as *GSSP5* > *GSSP6* > *GSSP7* > *GSSP3* > *GSSP1*; in stem, they were ranked as *GSSP6* > *GSSP5* > *GSSP7* > *GSSP3* > *GSSP1*. These synthetic promoters showed different expression efficiencies in various green tissues: *GSSP3* and *GSSP5* showed higher expression efficiencies than the positive control (CaMV *35S*) in leaf, especially *GSSP3*, whose expression efficiency was 1.5-fold that of the positive control; *GSSP1* and *GSSP5* showed high expression efficiencies in sheath; while *GSSP5* and *GSSP6* showed high expression efficiencies in panicle; and only *GSSP6* showed high expression efficiency in stem (Fig. 3). Therefore, these green tissue-specific synthetic promoters can be used for realizing efficient expression of different target genes to meet the requirements of various applications. Moreover, they overcome the defects of original regulatory sequences in tissue-specific expression: *P*_*Osrbcs-62*_ had no activity in stem^32^, while *GSSP6*, in which the GT1 and the first intron of rice *Act1* were respectively added to the upstream and downstream of *P*_*Osrbcs-62*_, showed sharply increased activity in stem; *P*_*D540-544*_ had activity not only in green tissues but also in root^7^, while *GSSP5*, in which *P*_*Osrbcs-550*_ and the first intron of rice *Act1* were inserted to the downstream of *P*_*D540-544*_, abolished the activity of *P*_*D540-544*_ in root.

### Functions of tissue-specific expression related regulatory sequences in synthetic promoters

*P*_*Osrbcs-550*_ has been reported to be a truncated green tissue-specific promoter^32^. According to Fig. 3, by comparing the GUS activity in leaf between *GSSP2* and *GSSP8* as well as between *GSSP3* and *GSSP7* transgenic plants, we found that when *P*_*Osrbcs-550*_ was used to replace *P*_*D540-544*_, the expression efficiency of the synthetic promoter in leaf was obviously increased. In addition, compared with that of the plants containing *GSSP1*, the GUS activity of *GSSP3, GSSP5* and *GSSP8* transgenic plants was more than twice in leaf. Based on the structures of the synthetic promoters, *GSSP3, GSSP5* and *GSSP8* contained both *P*_*Osrbcs-550*_ and the first intron of rice *Act1*, while *GSSP1* did not contain the first intron of rice *Act1*. Hence, it can be inferred that the coexistence of *P*_*Osrbcs-550*_ and the first intron of rice *Act1* can sharply increase the activity of synthetic promoters in leaf. According to the GUS activity of *GSSP1, GSSP3, GSSP5* and *GSSP8* transgenic plants, the synthetic promoters containing *P*_*Osrbcs-550*_ showed quite low expression efficiencies in stem. It can be inferred that *P*_*Osrbcs-550*_ has suppressive effects on the activity of synthetic promoters in stem, suggesting that *P*_*Osrbcs-550*_ contains the *cis*-elements which inhibit promoter activity in stem.

*P*_*Osrbcs-62*_, a further truncated version of *P*_*Osrbcs-550*_, had no activity in stem^32^. However, if it was placed between green tissue-specific *cis*-elements and the first intron of rice *Act1* (*GSSP4* and *GSSP6*), the synthetic promoters showed high expression efficiencies in stem. Compared with the plants containing any synthetic promoters with *P*_*Osrbcs-550*_ (*GSSP1, GSSP3, GSSP5* and *GSSP8*), *GSSP4* and *GSSP6* transgenic plants showed much higher GUS activity in stem. The above results indicate that although *P*_*Osrbcs-62*_ had no activity in stem (ie. *P*_*Osrbcs-62*_ contained no *cis*-element which provided promoter activity in stem), the suppressive effect of *P*_*Osrbcs-550*_ on promoter activity in stem was also eliminated when being truncated to *P*_*Osrbcs-62*_, suggesting that the *cis*-elements which inhibit promoter activity in stem are present in the region between *P*_*Osrbcs-550*_ and *P*_*Osrbcs-62*_.

*EnP3-110* has been reported to be a short green tissue-specific promoter^31^. By comparing the GUS activity in *GSSP2* and *GSSP7* transgenic plants, we found that when *EnP3-110* was added in the synthetic promoter, the expression level of the target gene in sheath, stem and root was sharply increased.

*P*_*D540-544*_ was an expression regulatory sequence which had activity in green tissues as well as in root^7^. GUS activity in the root of *GSSP1, GSSP5* and *GSSP7* transgenic plants indicated that the activity of *P*_*D540-544*_ in root can be abolished by *P*_*Osrbcs-550*_ or the first intron of rice *Act1* adjacent to its downstream. The comparison of GUS activity in panicle between *GSSP3* and *GSSP5* transgenic plants indicated that when *P*_*D540-544*_ was added in the synthetic promoter, the expression level of the target gene in panicle was increased. However, the plants containing *GSSP1*, which was only composed of *P*_*D540-544*_ and *P*_*Osrbcs-550*_, showed quite low GUS activity in panicle. These results indicate that the coexistence of *P*_*D540-544*_ and the first intron of rice *Act1* can dramatically increase the activity of synthetic promoters in panicle.

The G Box has been reported as a *cis*-element related to green tissue expression^30^. However, by comparing GUS activity in *GSSP4* and *GSSP6* transgenic plants, we found that when the G Box was placed in the upstream of ‘*P*_*Osrbcs-62*_ + the first intron of rice *Act1*’, activity of the synthetic promoter in non-green tissues embryo and root was observed. If GT1 was used to replace G Box, the expression pattern of the synthetic promoter could be restored to green tissue-specificity and the expression efficiency of the promoter in sheath and panicle was also obviously increased.

The first intron of rice *Act1* is an expression regulatory sequence, which can increase the activity of adjacent promoter^29^. The above results indicate that the coexistence of the first intron of rice *Act1* and *P*_*Osrbcs-550*_/*P*_*D540-544*_ can greatly increase the activity of the synthetic promoter in leaf/panicle. Meanwhile, analysis of GUS activity in *GSSP1* and *GSSP5* transgenic plants suggested that the first intron of rice *Act1* can not increase the activity of the promoter in sheath, endosperm, embryo and root. These results imply that the increase of expression efficiency by the first intron of rice *Act1* shows some tissue-specificity, which might be related to the adjacent promoter^34^.

### Screening of *cis*-elements involved in green tissue-specific expression

**Figure 4:**
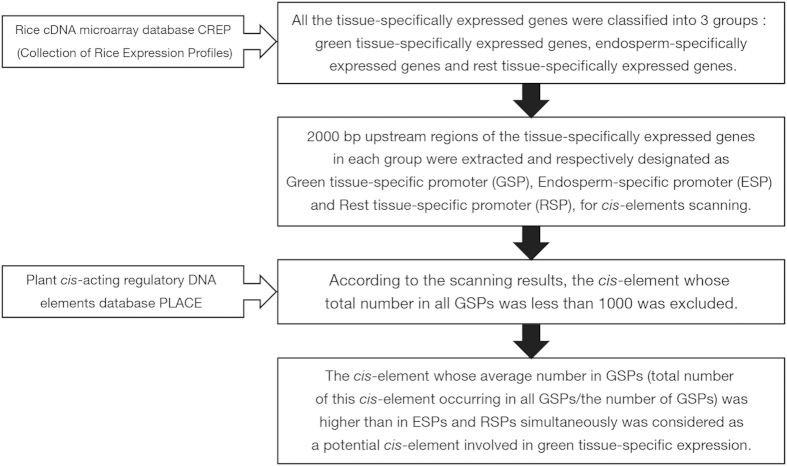
Bioinformatic identification of *cis*-elements involved in green tissue-specific expression.

According to the method described in Fig. 4, 10 potential *cis*-elements involved in green tissue-specific expression were obtained (Table 1). The frequencies and total numbers of these *cis*-elements in bioinformatic identification are shown in Table 2. Among them, GT1 and GATABOX are known *cis*-elements for light-regulation and green tissue-specific expression^30^,^33^; CACTFTPPCA1 is involved in mesophyll-specific expression^35^; MYCCONSENSUSAT was reported to be involved in green tissue-specific expression and cold-induction^36^; and WBOXNTERF3 is related to the activation of gene expression by wounding in leaf^37^. Besides, RAV1AAT might be related to high expression of transcription factor in leaf and root^38^. Half of the 10 candidate *cis*-elements have been reported to be involved in green tissue expression, which proves the availability of our method. In order to find a novel *cis*-element involved in green tissue-specific expression, core element ROOTMOTIFTAPOX1 (5′-ATATT-3′, here designated as GEAT which stands for Green tissue-specifically Expressed AT-rich element), which is not related to green tissue-specific expression based on the existing annotation, was selected for further study.

### Bioinformatic analysis, identification and site-directed mutagenesis of GEAT flanking sequence

**Figure 5:**
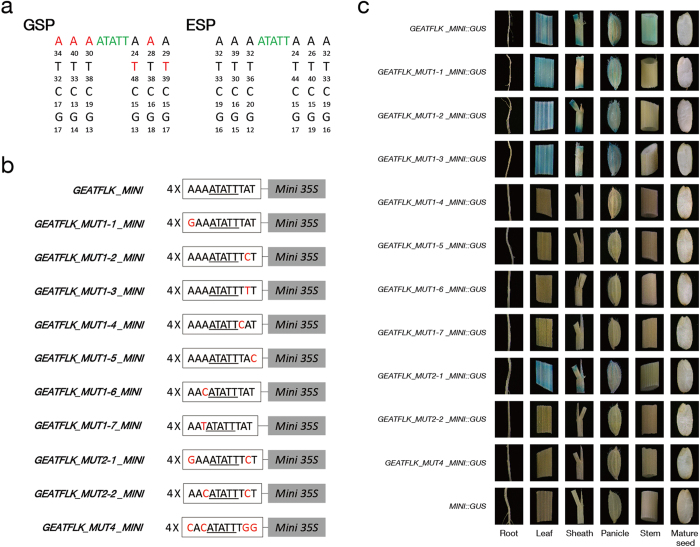
Bioinformatic and experimental analysis of GEAT flanking sequence. (**a**) Frequencies (%) of GEAT flanking sequence bases (3 left and 3 right) in GSPs and ESPs, respectively. Red letters indicate the bases chosen as optimal bases. (**b**) Schemes of synthetic promoters for functional identification of GEAT with its flanking sequence. Tetramer of GEAT and its flanking sequence (4 × GEATFLK / 4 × GEATFLK_MUT) was placed upstream of −46 Minimal *35S*. The underlined sequences indicate the core element GEAT. Mutation sites are indicated by red letters. (**c**) Histochemical analysis of *GUS* expression in transgenic plants for functional identification of GEAT with its flanking sequence. *MINI::GUS*, negative control, plants containing −46 Minimal *35S*-pDX2181.

According to the scanning and statistical results of GEAT in GSPs and ESPs (Fig. 5a), GEAT and its flanking sequence (GEATFLK) was determined as 5′-A_1_A_2_A_3_ATATTT_4_A_5_T_6_-3′ (The underlined sequence indicates GEAT). The method for the determination of GEAT flanking sequence was described in Methods. As the presence of TATABOX-like sequence led to the increase of frequency of T at the third site, A was set as the optimal base of the third site in order to avoid interference. Tetramer of GEATFLK was placed upstream of -46 Minimal *35S* to promote the expression of *GUS* (Fig. 5b). The results of GUS assays in transgenic plants showed that the target gene was specifically expressed in leaf, sheath, panicle and stem (Fig. 5c). Thus, it can be confirmed that we successfully identified a novel green tissue-specific *cis*-element (GEAT) with its flanking sequence.

Flanking sequence may influence the activity of core element^17^. Therefore, the function of GEAT flanking sequence was further analyzed by mutation assays (Fig. 5b). According to the results of GUS assays in single mutation transgenic plants, mutation at any of A_3_, T_4_ and T_6_ could completely abolish the activity of GEAT, indicating that A_3_, T_4_ and T_6_ are critical for the function of GEAT. Mutation at A_1_ or A_5_ could not eliminate the activity of GEAT in green tissues except for the stem, in which GEAT activity was lost (Fig. 5c). It can be inferred that A_1_ and A_5_ are important for maintaining the activity of GEAT in stem, and mutation at any of them can change the functional pattern of GEAT.

Since GEAT with single mutation at A_1_ or A_5_ still had activity in green tissues except for the stem, A_1_ and A_5_ were mutated simultaneously to find if double mutation can influence the functions of GEAT in other tissues. The results indicate that double mutation at A_1_ and A_5_ of GEAT resulted in the same expression pattern of the target gene with single mutation at A_1_ or A_5_. Hence, it can be inferred that multiple mutations at different flanking bases which have similar effects on GEAT can not produce a different functional pattern of GEAT with single mutation at one of these bases. As the mutation at A_5_ could still maintain the activity of GEAT but the mutation at A_3_ could not, A_3_ and A_5_ were mutated simultaneously to find out whether mutation at A_5_ can restore the activity abolished by mutation at A_3_. The result indicated that the activity of GEAT can not be restored by mutation at A_5_. Therefore, it can be inferred that the mutation at any critical base results in an irreversible abolishment of GEAT activity, which can not be restored by mutation at other bases. Finally, the result of quadruple mutation in GEAT was consistent with our anticipation: when A_1_, A_3_, A_5_ and T_6_ were mutated simultaneously, the activity of GEAT was completely abolished.

According to the above results, the flanking sequence which supports the activity of GEAT was identified as 5′-AAAATATTTAT-3′ (the dotted bases are critical for maintaining the function of GEAT).

## Discussion

In this study, we fused several regulatory sequences related to tissue-specific expression in 8 different combinations. The GUS assays of transgenic plants confirmed that we successfully created 5 green tissue-specific synthetic promoters and proved the feasibility of synthesizing tissue-specific promoters in rice as well. Meanwhile, these novel synthetic promoters can overcome the defects of original regulatory sequences in tissue-specific expression. They also showed different expression efficiencies in various green tissues and thus can meet the requirements of various applications. *GSSP3* showed the highest expression efficiency in leaf, which was 1.5-fold that of the positive control. Therefore, it can be applied to transgenic breeding for improving disease/pest resistance in rice leaf^28^,^39^, as well as to the studies of the genes related to leaf senescence and other leaf traits^40^. *GSSP5* showed high expression efficiency in leaf, sheath and panicle. Hence, it can be used for efficient expression of the genes related to blast resistance in rice^41^ as well as for the studies of photosynthesis-related genes^42^. Although *GSSP2* had activity in root, it showed the highest expression efficiency in sheath and stem among all the synthetic promoters and had no activity in endosperm and embryo. Therefore, it can be used for efficient expression of target genes for resistance to pest (such as striped stem borer, brown planthopper and rice plant weevil) and disease (such as rice sheath blight) in rice^43^,^44^,^45^,^46^, and it is also helpful to the studies of height-related genes^47^.

Subsequently, the functions of these expression regulatory sequences in synthetic promoters were analyzed. The results are highly valuable for the theoretical and applied research of synthetic promoters. For example, ‘*P*_*Osrbcs-550*_ + the first intron of rice *Act1*’ or ‘*P*_*D540-544*_ + the first intron of rice *Act1*’ can be added to the target promoter to achieve a great increase of promoter activity in leaf or in panicle, respectively; *EnP3-110* can be added to increase promoter activity in sheath, stem and root; and ‘GT1 + *P*_*Osrbcs-62*_ + the first intron of rice *Act1*’ can be used to enhance promoter activity in sheath, stem and panicle. However, some functions of these expression regulatory sequences were different from those in previous reports. For example, *P*_*Osrbcs-550*,_ *EnP3-110* and G Box are green tissue-specific regulatory sequences. However, *P*_*Osrbcs-550*_shows suppressive effects on the synthetic promoter activity in stem; *EnP3-110* can greatly increase promoter activity in root; and when G Box is placed in the upstream of ‘*P*_*Osrbcs-62*_ + the first intron of rice *Act1*’, it may increase promoter activity in non-green tissues embryo and root. These instances may arise from the interactions of *cis*-elements and should be explored in future studies.

*Cis*-element is an essential part of the synthetic promoter. As the feasibility of synthesizing tissue-specific promoters in rice was proved, an available method for the discovery of tissue-specific *cis*-elements is significant to the development of tissue-specific synthetic promoters. Therefore, another major aim of the present study was to screen and identify *cis*-elements related to green tissue-specific expression. With the screening method designed in this study, we obtained 10 potential *cis*-elements involved in green tissue-specific expression based on the information from the rice cDNA microarray database CREP. Half of the 10 candidate *cis*-elements have been reported to be involved in green tissue expression, which proves the availability of our method. In order to find a novel *cis*-element involved in green tissue-specific expression, a core element ROOTMOTIFTAPOX1, which is not related to green tissue-specific expression, was chosen and named as GEAT for further study. The results of GUS assays in *GEATFLK_MINI::GUS* transgenic plants showed that the target gene was specifically expressed in green tissues. This result demonstrates that we successfully identified a novel green tissue-specific expression related *cis*-element GEAT with its flanking sequence. Moreover, it further proves the reliability of this screening method.

As flanking sequence may influence the activity of core element^17^, the function of GEAT flanking sequence was analyzed specifically in this work. We found several bases which were indispensable to the whole function or functional pattern of GEAT: A_3_, T_4_ and T_6_ are critical for the whole function of GEAT, and mutation at any of them can completely abolish the activity of GEAT; A_1_ and A_5_ are indispensable for the activity of GEAT in stem, and mutation at either of them can change the functional pattern of GEAT. Furthermore, based on the results of double mutation, we found that multiple mutations at different flanking bases which have similar effects on GEAT can not produce a different functional pattern of GEAT with single mutation at one of these bases, and mutation at a critical base will irreversibly abolish the activity of GEAT.

According to the statistical analysis of flanking bases, the frequency of T_4_ was 44% in ESPs, while it reached up to 48% in GSPs. This result suggests that T_4_ is important to GEAT, and is even more important to the function of GEAT in GSPs. Mutation assays also proved that it is indispensable for the function of GEAT. T_6_ showed similar characteristics with T_4_: its frequency was 33% in ESPs, and was 39% in GSPs. Our results also indicate that when T_6_ is mutated, GEAT will completely lose its activity. These results verify the reliability of our method for flanking sequence analysis. We did not perform mutation assay at A_2_ because whatever bases it was mutated to, several additional expression-promoting *cis*-elements will be formed in the mutant. Based on its frequency (39% in ESPs and 40% in GSPs), we infer that A_2_ may play a role in the function of GEAT.

The original annotation of GEAT is a *cis*-element related to gene expression in root^48^. However, the previous study only predicted its function with bioinformatic analysis, and did not identify this *cis*-element with transgenic approach. Besides, for the reason that flanking sequence may influence the activity of the core element^17^, we speculate that even if GEAT can function in root, its activity still needs the support from some specific flanking sequences. In this work, when we mutated A_1_ or A_5_, GEAT could not maintain its activity in stem and the expression pattern of the target gene was changed, which also supports our hypothesis.

The traditional experimental approach for seeking and identifying *cis*-elements is mainly based on electrophoretic mobility shift assay (EMSA). However, either the weak binding capacity of *cis*-element and TFs or the low content of the target TFs in nuclear proteins may lead to the dissociation of the complex in gel electrophoresis. Besides, even if the TFs-binding activity of a *cis*-element has been proved, it remains unknown whether its interaction with TFs activates or represses transcription of genes, and whether the interaction functions constitutively or acts in specific tissues and stages. Therefore, it is still necessary to identify the *cis*-element with transgenic approaches. There are several instances in our study showing that some active *cis*-elements can not drive the expression of the target gene. For example, both TATABOX and SEF1MOTIF in *GEATFLK_MUT1-6_MINI* and *GEATFLK_MUT1-7_MINI* have been proved to possess TFs-binding activity^49^,^50^, but even with these *cis*-elements, *GEATFLK_MUT1-6_MINI* and *GEATFLK_MUT1-7_MINI* still had no activity. Meanwhile, when A_1_ or A_5_ was mutated, GEAT still showed activity in green tissues except for the stem, suggesting that even if the *cis*-element has TFs-binding and expression promoting activity, it is still uncertain whether the function pattern of the *cis*-element is changed. Therefore, EMSA is not sufficient to clarify the functions of *cis*-elements in gene expression. In this study, we initially applied synthetic promoters for the identification of tissue-specific *cis*-elements. This approach overcomes the limitation of EMSA in *cis*-element analysis and has been successfully applied to identify the function patterns of *cis*-elements combined with different flanking sequences. Overall, we obtained 5 novel green tissue-specific synthetic promoters which can be widely applied in genetic engineering, and provided an example for the synthesis of tissue-specific promoters in rice. We also developed a feasible method for screening and functional identification of tissue-specific *cis*-elements with their flanking sequences at the genome-wide level in rice.

## Methods

### Synthetic promoters vector construction

Sequences of *P*_*D540-544*_, *P*_*Osrbcs-550*_, *P*_*Osrbcs-62*_, *EnP3-110*, the first intron of rice *Act1*, G box and GT1 used here were the same as previous reports^7^,^29^,^30^,^31^,^32^,^33^ (see Supplementary Fig. S1). The above regulatory sequences were respectively derived from: LOC_Os08g10020 (*P*_*D540-544*_), LOC_Os12g17600 (*P*_*Osrbcs-550*_), LOC_Os12g17600 (*P*_*Osrbcs-62*_), LOC_Os03g55734 (*EnP3-110*) and LOC_Os03g50885 (the first intron of rice *Act1*). All of the regulatory sequences above were used to synthesize promoters, and their schemes are shown in Fig. 1. These constructs were synthesized by GenScript and ligated into the promoter functional analysis vector pDX2181 after digesting with *Hind* III and *Pst* I^11^.

### *Agrobacterium*-mediated transformation to rice callus

The sequence-confirmed clones were transformed into the *Agrobacterium tumefaciens* strain *EHA105* by electroporation. Subsequently, all the constructs were introduced into Zhonghua11 (*Oryza sativa* L. ssp. *japonica*) by *Agrobacterium*-mediated transformation. pDX2181 (the negative control) and CaMV *35S*-pDX2181 (the positive control) were also introduced into Zhonghua11 in the same way. The callus culture and transformation procedures were carried out as previously described^51^.

### Histochemical and fluorometric analysis of GUS activity

Histochemical staining of GUS activity in rice tissues was conducted essentially as described previously^52^. Various tissues of T_0_ transgenic-positive transformants (root, leaf, sheath, panicle, stem and mature seed) were incubated in GUS staining solution (50 mM sodium phosphate at pH 7.0, 10 mM Na_2_-EDTA, 0.1% Triton X-100, 1 mg/mL X-Gluc, 100 μg/ml chloramphenicol, 1 mM potassium ferricyanide, 1 mM potassium ferrocyanide and 20% methanol) at 37 °C for 2–10 h after 15-min vacuum filtration. After GUS staining, the samples were incubated in 70% ethanol to remove chlorophyll and photographs were taken under a dissecting microscope (Leica MZFLIII).

Quantitative analysis of GUS activity was conducted as previously described^53^. The total protein concentration in the supernatant was quantified using the Bradford assay^54^. GUS protein in the supernatant was determined fluorometrically with an INFINITE 200 photometer (Tecan Austria Gmbh, Ltd, Grodig, Austria). GUS activity was determined fluorometrically by measuring the amount of 4-methylumbelliferone (Mu) produced under the catalysis of GUS in 1 mg of total protein per minute.

### Screening of *cis*-elements involved in green tissue-specific expression

The information of various tissue-specifically expressed genes in rice was derived from the rice cDNA microarray database CREP (Collection of Rice Expression Profiles, http://crep.ncpgr.cn)^23^. All the tissue-specifically expressed genes can be divided into 2 groups based on their expression patterns: green tissue-specifically expressed genes (expressed only in green tissues, such as shoot, leaf, sheath, spikelet, panicle (stage 5) and stem) and other tissue-specifically expressed genes. However, although other tissues are not green tissues, part of them are related to green tissues to some extent, such as panicle (stages 1–4) and plumule^23^. Among the identified tissues in this study, endosperm has no relationship with green tissues and the number of endosperm-specifically expressed genes is sufficient to exclude the influence of the random arrangement of bases in *cis*-elements analysis. Therefore, we separated endosperm-specifically expressed genes from other tissue-specifically expressed genes as an independent control in order to avoid the interference from numerous non-green tissue-specifically expressed genes. Hence, we divided all the tissue-specifically expressed genes into 3 groups: green tissue-specifically expressed genes (n = 210), endosperm-specifically expressed genes (n = 164) and rest tissue-specifically expressed genes (n = 1019). 2000 bp upstream regions of these genes were extracted and set as promoters for analysis of *cis*-elements, which were designated as Green tissue-specific promoter (GSP), Endosperm-specific promoter (ESP) and Rest tissue-specific promoter (RSP), respectively. Based on the information of *cis*-elements in PLACE database^55^, frequencies of various *cis*-elements in GSPs, ESPs and RSPs were scanned and subsequently counted. According to the results, the total number of a single *cis*-element occurring in all GSPs ranged from 1 to 6000. In order to exclude the influence of random events, the *cis*-element whose total number in all GSPs was less than 1000 was abandoned. Among the rest *cis*-elements, the one whose average number in GSPs (total number of this *cis*-element occurring in all GSPs/the number of GSPs) was simultaneously higher than in ESPs and RSPs was considered as a potential *cis*-element involved in green tissue-specific expression (Fig. 4).

### Bioinformatic analysis, identification and site-directed mutagenesis of GEAT flanking sequence

GEAT flanking sequence composed of six ‘optimal bases’ (3 left and 3 right) was determined based on the scanning and statistical results of GEAT in GSPs and ESPs. A GEAT flanking sequence base was set as an optimal base if its frequency at one site in GSPs was higher than at the corresponding site in ESPs and was also higher than 25%.

Tetramer of GEAT and its flanking sequence (4 × GEATFLK) was placed upstream of −46 Minimal *35S* to promote the expression of *GUS* (*GEATFLK_MINI::GUS*) (Fig. 5b). Under the premise of no formation of additional expression-promoting *cis*-elements, the flanking sequence of GEAT was treated with single, double and quadruple mutation. Tetramer of GEAT and its mutant flanking sequence was placed upstream of −46 Minimal *35S* to promote the expression of *GUS* (*GEATFLK_MUT_MINI::GUS*) (Fig. 5b). All the constructs above and −46 Minimal *35S*-pDX2181 (*MINI::GUS*, the negative control) were transformed into Zhonghua11, respectively. Vector construction, callus culture and transformation as well as histochemical staining of GUS activity were performed as described above.

## Additional Information

**How to cite this article**: Wang, R. *et al.* Novel green tissue-specific synthetic promoters and *cis*-regulatory elements in rice. *Sci. Rep.* **5**, 18256; doi: 10.1038/srep18256 (2015).

## Supplementary Material

Supplementary Information

## Figures and Tables

**Table 1:** Sequences and annotations of 10 potential green tissue-specific *cis*-elements.

***Cis*****-elements**	**Sequence**	**Existing Annotation**
GATABOX^30^	GATA	Involved in light-regulation and green tissue-specific expression
GT1^33^	GRWAAW	Involved in light-regulated expression
CACTFTPPCA1^35^	YACT	Involved in mesophyll-specific expression
MYCCONSENSUSAT^36^	CANNTG	Involved in leaf and silique-specific expression and cold-induction
WBOXNTERF3^37^	TGACY	Involved in activation of gene expression by wounding in tobacco leaf
ROOTMOTIFTAPOX1^48^	ATATT	Involved in gene expression in root
RAV1AAT^38^	CAACA	Binding site of RAV1, which were highly expressed in rosette leaf and root
ARR1AT^56^	NGATT	Involved in activation of gene expression by the cytokinin-regulated transcription factor
WRKY71OS^57^	TGAC	Binding site of rice WRKY71, a transcriptional repressor of the gibberellin signaling pathway
CURECORECR^58^	GTAC	Involved in copper- and oxygen-response

*Cis*-elements which have been reported to be involved in green tissue-specific expression are indicated in bold.

**Table 2:** Average numbers (ANs) in GSPs, ESPs and RSPs and total numbers (TNs) in GSPs of 10 potential green tissue-specific *cis*-elements.

***Cis*****-elements**	**ANs**			**TNs**
	**GSPs**	**ESPs**	**RSPs**	
CACTFTPPCA1	28.5	26.9	25.9	5980
ARR1AT	17.9	16.3	16.5	3749
MYCCONSENSUSAT	16.9	15.9	15.7	3556
GT1	14.7	13.9	13.5	3078
GATABOX	14.6	13.9	13.3	3073
WRKY71OS	12.7	11.7	11	2658
CURECORECR	11.3	10.6	10.2	2366
ROOTMOTIFTAPOX1	10.9	9.1	9.3	2296
WBOXNTERF3	6.4	5.3	5.0	1337
RAV1AAT	5.3	4.9	4.8	1111

(AN of a *cis*-element in GSPs = TN of this *cis*-element occurring in all GSPs/the number of GSPs).
