# Supplementary material for: Novel green tissue-specific synthetic promoters and cis-regulatory elements in rice
Source: Sci Rep. 2015 Dec 11;5:18256. doi: 10.1038/srep18256 (PMC4676006; doi:10.1038/srep18256)
Supplement: Supplementary Information [file srep18256-s1.pdf]

Supplementary information

Novel green tissue-specific synthetic promoters and *cis*-regulatory elements in rice

Rui Wang[1], Menglin Zhu[1], Rongjian Ye[1], Zuoxiong Liu[2], Fei Zhou[1], Hao Chen[1] and Yongjun Lin[1]*

1. *National Key Laboratory of Crop Genetic Improvement and National Centre of Plant Gene Research, Huazhong Agricultural University, Wuhan, China*

2. *College of Foreign Language, Huazhong Agricultural University, Wuhan, China*

* Correspondence (Tel: 86-027-87281719, Email: yongjunlin@mail.hzau.edu.cn)

$P_{D540\text{-}544}$ (585bp)

5’-TAGATAATGGATTAAAACCTGGCCTCTATATCCAAACTCGGATGTACATGGCCAAATT
AACATCCAGTTCTTCATCCACCTTCATATCAGAACATTCCAGAATACAGATTGCAATGG
CATCCATTGGCACCACATGTTTTGACTGATTCTTTCACTGTTTCTTGATATATTTGTTGAT
TTTTAATGGTAAATAGCTCATCTGTCCAATCCTGGCACCACATAATTTCCCCTGTTAAAA
CAGTGCCAACACAAATTTACTCATGTCCACTGAAAATCCCCAGAGGATAACGTTCTGG
CACTGCCATATCAGGACCGGACCATACGCGGCGCGCCACGTGGCGCGCCGGCGGTCAC
ACCTGTGCTGGTGCACCCTGGCATTATCCCCATCCACCATCCTGTGGGTGACTGGCCAC
CAAAGGTTTTTGGCGCCACAATTATATTGAGCTGCCATTGCTTCTCACCTCTGCTTTGCA
TCCATCCATCCATCAGAGATCAGGTAGAACAGTGAGAGTGAGGTGCAGAAAAATTTGA
GCTGAAGCTGAGGATGGCAACATCCATGATCACCTCGCCGCTGGTGGCGCCGGC-3’

GATABOX GT1CONSENSUS CACTFTPPCA1
SORLIP1 GBOXLERBCS SORLIP5

$P_{Osrbcs\text{-}550}$ (551bp)

5’-GCTTAATGAGGGCCCAAAGTTTTGATGACCTTTTGCTTGATCTCGAAATTAAAATTC
AAGTACCTGTTAAGGGAGGTCACACCACCATCAATTTTCAGCCTGAAGAAACAGTTAA
ACAACGACCCCGATGACCAGTCTACTGCTCTCCACATACTAGCAGCATTATTGATCACA
AAAGAAACCAAAATAAAAATCAGCACCGAGTGTGCAGAGGGAGACAAAGGTGATCTG
GCAGTGGATATCTCCCCATCCATCCTCACCCGCGCTGCCCATCACTCGCCGCCGCATACT
ACATCATGTGGAGAGAGGAAGACGAGGACCACAGCCAGAGCCCGGGTCGAGATGCCA
CCACGGCCACAATCCACGAGCCCGGCGCGACACCACCGCGCGCGCGTGAGCCAGCCA
CAAACGCCCGCGGATAGGCGCGCGCACGCCGGCCAATCCTACCACATCCCCGGCCTCC
GCGGCTCGCGAGCGCCGCTGCCATCCGATCCGCTGAGTTTTGGCTATTTATACGTACCG
CGGGAGCCTGTGTGCAGAGCAGTGCATCT-3’

CACTFTPPCA1 GATABOX SORLIP1
$P_{Osrbcs\text{-}62}$

EnP3-110 (110bp)

5’-CCTATAAATAATCCCCTAGAGCAATTGTTATCTCATCACCCTCAACATATAATCTTCTA
CATTTACACCATCTAGTAATCTTGTTAAGCATCTCCCATACGCTGTCAACA-3’

The first intron of rice *Act1* (314bp)

5’-GTAACCACCCCGCCCCTCTCCTCTTTCTTTCTCCGTTTTTTTTTTCGTCTCGGTCTCGA
TCTTTGGCCTTGGTAGTTTGGGTGGGCGAGAGCGGCTTCGTCGCCCAGATCGGTGCGC
GGGAGGGGCGGGATCTCGCGGCTGGCGTCTCCGGGCGTGAGTCGGCCCGGATCCTCG
CGGGGAATGGGGCTCTCGGATGTAGATCTTCTTTCTTTCTTCTTTTTGTGGTAGAATTTG
AATCCCTCAGCATTGTTCATCGGTAGTTTTTCTTTTCATGATTTGTGACAAATGCAGCCT
CGTGCGGAGCTTTTTTGTAG-3’

G box (12bp)

5’-TGACACGTGGCA-3’

GT1 (15bp)

5’-TGTGTGGTTAATATG-3’

Figure S1. Sequences and green tissue expression related *cis*-elements of the expression regulatory sequences used for designing synthetic promoters. (*EnP3-110* minimal promoter and the first intron of rice *Act1* did not contain upstream *cis*-elements related to green tissue-specific expression.)
